# Supplementary material for: Development of a computer-aided design software for the quantitative evaluation of aesthetic damage
Source: PLoS One. 2019 Dec 18;14(12):e0226322. doi: 10.1371/journal.pone.0226322 (PMC6919621; doi:10.1371/journal.pone.0226322)
Supplement: S1 Code site — 1. (ZIP) [file pone.0226322.s001.zip › [EESC_jr][Projeto]Relatorio_07_21/reports/templates_zip/gentelella-master/production/map.html]

Gentellela Alela! | 


Gentellela Alela!

Welcome,

## John Doe

  


### General

- Home 
  - Dashboard
  - Dashboard2
  - Dashboard3
- Forms 
  - General Form
  - Advanced Components
  - Form Validation
  - Form Wizard
  - Form Upload
  - Form Buttons
- UI Elements 
  - General Elements
  - Media Gallery
  - Typography
  - Icons
  - Glyphicons
  - Widgets
  - Invoice
  - Inbox
  - Calendar
- Tables 
  - Tables
  - Table Dynamic
- Data Presentation 
  - Chart JS
  - Chart JS2
  - Moris JS
  - ECharts
  - Other Charts

### Live On

- Additional Pages 
  - E-commerce
  - E-commerce Backend
  - Projects
  - Project Detail
  - Contacts
  - Profile
  - Real Estate
- Extras 
  - 403 Error
  - 404 Error
  - 500 Error
  - Coming Soon
  - Plain Page
  - Login Page
  - SignUp Page
  - Pricing Tables
- Landing Page Coming Soon

- - John Doe
    - Profile
    - 50%
      Settings
    - Help
    - Log Out
  - 6
    - John Smith
      3 mins ago

      Film festivals used to be do-or-die moments for movie makers. They were where...
    - John Smith
      3 mins ago

      Film festivals used to be do-or-die moments for movie makers. They were where...
    - John Smith
      3 mins ago

      Film festivals used to be do-or-die moments for movie makers. They were where...
    - John Smith
      3 mins ago

      Film festivals used to be do-or-die moments for movie makers. They were where...
    - **See All Alerts**
- - Action
  - Another action
  - Something else here
  - Separated link


SixxmReg Dashboard


SimReg Dashboard
